# Dual Functions in Response to Heat Stress and Spermatogenesis: Characterization of Expression Profile of Small Heat Shock Proteins 9 and 10 in Goat Testis

**DOI:** 10.1155/2015/686239

**Published:** 2015-01-22

**Authors:** Wenjuan Xun, Liguang Shi, Ting Cao, Chunping Zhao, Ping Yu, Dingfa Wang, Guanyu Hou, Hanlin Zhou

**Affiliations:** Tropical Crop Genetic Resource Research Institute, Chinese Academy of Tropical Agricultural Sciences (CATAS), Danzhou, Hainan 571737, China

## Abstract

Small heat shock proteins 9 and 10 (HSPB9 and HSPB10) are two testis-specific expressed sHsps. The objective of this study was to investigate the mRNA expression profile of HSPB9 and HSPB10 in goat testis among the different seasons, ages, and environmental temperatures. Allocation of the two sHsps was also performed by immunohistochemistry. The results showed that the transcript levels of HSPB9 and HSPB10 were extremely high in the testis (*P* < 0.01). The relative expression of HSBP9 and HSPB10 in testis showed a tendency to increase with age and then is maintained at the constant level after sexual maturity. HSPB9 and HSPB10 have significantly higher expression in the breeding season  (*P* < 0.05) and hot season (*P* < 0.01). Both HSPB9 and HSPB10 were found to be upregulated by high-temperature stress in testis (*P* < 0.05), and the expressions of Hsp70 and Hsp90 were also increased simultaneously (*P* < 0.01). Immunohistochemistry analysis localized HSPB9 expressed in spermatogonia, spermatocytes, and round spermatids and HSPB10 expressed in the elongate spermatids. In epididymis, strongly staining signal of HSPB10 was detected in pseudostratified columnar epithelium. In conclusion, the two testis-specific sHsps are closely related to male reproduction and heat tolerance. The results could provide valuable data for the further studies on HSPB9 and HSPB10.

## 1. Introduction

Heat shock proteins (Hsps) are thought of as chaperones of morphologic development in cells and organisms. They played important “housekeeping” role and were first discovered due to their increased expression in heat-shocked and stressed cells. According to their molecular weights, sHsps can be classified into seven major families: Hsp110/100, Hsp90, Hsp70, Hsp60, Hsp40, Hsp33, and small heat shock proteins (sHsps or HSPB) [1, 2]. Hsps also belong to the widely expressed chaperone proteins with molecular weights mass of 14 kDa to 30 kDa (the majority between 15 and 27 kDa). As the latest discovered Hsps, they have their own unique characteristics, such as conserved *α*-crystallin domain in the C-terminus [3], ATP-independent holdase activity [4], and required oligomeric complex structures for their activation [5]. Until now, at least ten different sHsps, several with tissue-restricted expression, are identified in higher eukaryotes. It has been demonstrated that the temporal- and tissue-specificities expressed sHsps are not only upregulated by noxious environmental stimuli such as hyperthermia stress, hypoxia, and harmful oxidants, but also involved in a variety of physiological functions in cytoskeletal rearrangements [6], fibril formation [7], apoptosis [8], and oxidation-reduction [9]. However, the mechanisms underlying the functions of sHsps are not well known.

HSPB9 and HSPB10 are two testis-specific expressed sHsps [10]. In human, HSPB9 is characterized by a continuous open reading frame, encoding a protein of 159 residues with the mass of 17.5 kDa [11]. As determined by northern blotting, HSPB9 is expressed exclusively in testis. It was also demonstrated that HSPB9 belongs to the steadily growing number of cancer/testis antigens, which interacts with the dynein subunit TCTEL1 [12]. In contrast with other sHsps, expression of HSPB9 in normal testis was specifically detected in pachytene spermatocytes and increased with age [13]. However, there is little recent literature on the properties and actual function of HSPB9 in spermatogenesis. HSPB10 or outer dense fiber protein 1 (ODF1), first reported in 1993 [14], has a molecular weight of 27 kDa with a high content of cysteine. It has a C-terminal tail which is similar to some keratins [15] and serves a structural role in the sperm tail [16]. It was also different with HSPB1 in which mRNA and protein could be detected in spermatocytes as well as in Sertoli cells reported by the previous studies [17, 18]. As a major protein of the mammalian sperm tail, HSPB10 was specifically found in the sperm tails of vertebrates but not in eukaryotic cilia and flagella [19]. Recently, some interesting reports on HSPB10 showed that it can be used as the sperm freezability markers [20]. Thus, it can be inferred that HSPB10 also participated in responding environment heat stresses. However, the investigation of functional characterization of HSPB10 was limited. The role in spermatogenesis is still an enigma to male fertility.

Goat (*Capra hircus*) is the important economic livestock in the world. In southern China, especially in Guangdong, Guangxi, and Hainan Province, goats are the most common shelter-feeding ruminants. A lot of research works have been achieved in the indigenous small ruminant in tropical areas, but limited studies focused on sHsp. In our previous research, the full-length HSPB9 and HSPB10 cDNA sequence of goat have been cloned (GenBank ID: JX088726; JX067553). In this study, we extensively determined the mRNA expression profile of HSPB9 and HSPB10 in the different seasons, developmental stages, and environmental temperatures in goat testis. Immunohistochemistry was also performed to visualize HSPB9 and HSPB10 in male gonad.

## 2. Materials and Methods

### 2.1. Geographical Location and Climate

The studies were carried out on the Hainan Black Goat Breeding Center of Chinese Academy of Tropical Agricultural Sciences (CATAS) in southern China (109.5°E, 19.5°N), which has a typical tropical climate all year round. The mean temperature of this area is 18°C in December and 29°C in July.

### 2.2. Animals and Sampling Procedures

All studies were conducted in compliance with the requirements of the Animal Ethics Committee of Chinese Academy of Tropical Agricultural Sciences. Animal experiments were done according to the guidelines for animal experiments at the National Institute of Animal Health. Totally 93 male Hainan black goats were included in this study. The diet was provided ad libitum as pelleted total mixed ration (TMR), which contained maize meal, soybean meal, wheat bran, sunflower seed meal, and vitamin and mineral supplements, respectively. The fresh potable water was available at all times. The testicular samples of thirty sexually mature Hainan black goats were harvested in different seasons (January, March, May, July, September, and November, resp., five animals for every separate time point) by castration under the local anesthesia. Eight adult goats (2 years old) were killed by exsanguinations, and the heart, kidneys, liver, lung, muscle, testis, cerebrum, epididymis, and spleen were collected. For age-dependent expression analysis of HSPB9 and HSPB10, forty male bucks aged 0 weeks, 1 week, 2 weeks, 4 weeks, 6 weeks, 8 weeks, 10 weeks, 12 weeks, 16 weeks, and 20 weeks were castrated (four animals for every separate time point). Another fifteen adult goats (2.5 years old) were fed in an environment-controlled room for 96 h (temperature about 18°C, 25°C, and 35°C, resp.). Five animals were used for every temperature. And then the testicular samples were castrated. All fresh tissue samples were immediately frozen in liquid nitrogen and stored at −80°C until analysis by real-time quantitative RT-PCR and other methods. The testis and epididymis were dissected into 0.5 cm × 0.5 cm × 0.2 cm and fixed in a 4% paraformaldehyde/PBS solution at 4°C for 24 h.

### 2.3. RNA Extraction and cDNA Synthesis

Total RNA was extracted by using the Trizol reagent (Invitrogen, USA) following the supplier's protocol. Total RNA was determined by running samples on 1% denaturing agarose gel and quantified by a spectrophotometer system (NanoDrop ND-1000, USA). Total RNA was cleaned up from contaminating DNA using RNeasy Mini Kit (Invitrogen, USA) following the manufacturer's instructions. Two-microgram total RNA was reverse-transcribed using Superscript system according to the manufacturer's directions (PrimeScript RT reagent Kit, TaKaRa). The cDNA templates were stored at −20°C for relative quantitative real-time PCR.

### 2.4. Real-Time Quantitative RT-PCR

Relative quantitative real-time PCR was used to analyze mRNA expression patterns of sHsps. The cDNA served as a template for the PCR amplification. The gene-specific primers were designed based on the sequence of goat HSPB9, HSPB10, Hsp70, and Hsp90 mRNA (GenBank ID: JX088726; JX067553; JN604433; and AF548366) and *β*-actin as a housekeeping gene based on *β*-actin mRNA (GenBank ID: JX046106) by using Primer Premier 3.0 online software. The sequences of these primers were as follows: HSPB9 (forward 5′-GTTGGTTCAGGTGAACAGCG-3′, reverse 5′-GGTCCGGTGGTAGTGACATT-3′); HSPB10 (forward 5′-AGCTTTACTGTCTTCGCCCG-3′, reverse 5′-CAGCAAGAGGACGCCAGAAT-3′); Hsp70 (forward 5′-CCCACGAAGCAGACGCAGAT-3′, reverse 5′-CCCAGCAGGTTGTTGTCCCG-3′); Hsp90 (forward 5′-ACTTGGCGGTCAAGCAT-3′, reverse 5′-ACTCTGCGAACATACAACT-3′); and *β*-actin (forward 5′-GGATGAGGCTCAGAGCAAGA-3′, reverse 5′-CGCAGCTCGTTGTAGAAGGT-3′). qRT-PCR reactions were performed on an Eppendorf Mastercycler ep realplex system (Eppendorf, Germany), and SYBR Green was used as the detection dye (SYBR Premix Ex Taq Kit, Takara) according to the manufacturer's instructions. The reaction was performed at 95°C for 10 s, 40 cycles of 95°C for 10 s, 62°C for 20 s, and a following cycle of 62°C for 30 s and 95°C for 15 s to obtain the dissociation curves. The uniform amplification of the products was verified by analyzing the melting curves of the amplified products in which melting temperatures (*T*_*m*_) were produced as follows: HSPB9, 85.5°C; HSPB10, 83.7°C; Hsp70, 88.1°C; Hsp90, 85.9°C; and *β*-actin, 80.5°C. The relative expression was evaluated with the 2^−ΔΔCT^ method [21]. Data of real-time PCR analysis subjected to ANOVA and *t*-test was used to determine the difference in mean values with SPSS software. The *P* value for significance was set at *P* < 0.05.

### 2.5. Immunohistochemistry

Fixed testis and epididymis samples were dehydrated in an upgraded series of ethanol (four times, 15 min each) and finally embedded in paraffin wax. Paraffin sections (4 *μ*m) were cut, disposed on glass slides, and dried overnight at 60°C. Serial paraffin sections were deparaffinized in xylene and rehydrated in decreasing concentrations of ethanol. Immunostaining was performed using the SABC kit according to the manufacturer instructions (BOSTER, Wuhan, China). To block endogenous peroxidase activity, slides were incubated with 3% H_2_O_2_ for 20 min. To prevent background staining, sections were incubated in normal goat serum at room temperature for 20 min after being washed in phosphate buffer saline (PBS, 0.01 M pH 7.2). Rabbit polyclonal anti-HSPB9 primary antibody (Abcam, USA) and mouse monoclonal anti-HSPB10 primary antibody (Santa Cruz, USA) were diluted 1 : 100 in PBS buffer (0.01 M, pH 7.2) and incubated overnight at 4°C. Thereafter, the sections were incubated with the polymerized HRP conjugated mouse-anti-rabbit or rabbit-anti-mouse IgG and streptavidin-biotin-complex (SABC) under the instruction of manufacturer. Immunoreaction products were visualized using 3,3′-diaminobenzidine (DAB) and counterstained with hematoxylin. Negative controls were performed in parallel under identical conditions, either by omitting the primary antibody or by replacing it with normal rabbit serum. Finally, the sections were imaged using a Leica DMIRB microscope (Leica, Germany).

## 3. Results

### 3.1. Expression of HSPB9 and HSPB10 mRNA in Various Tissues

**Figure 1:**
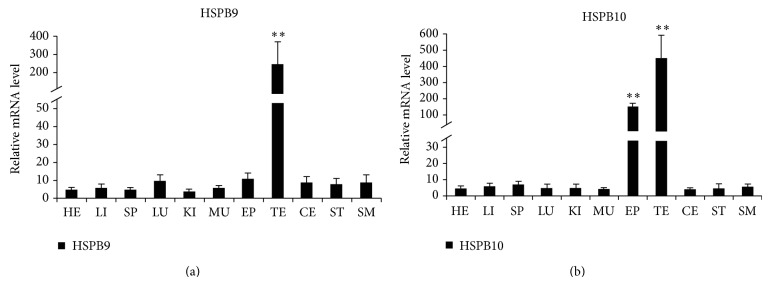
mRNA expression profile of HSPB9 and HSPB10 in adult goat tissues. (a) Tissue distribution of HSPB9 mRNA in goat. (b) Tissue distribution of HSPB10 mRNA in goat. Five independent reactions were performed per sample, and the mean values of 8 samples (mean ± SD, *n* = 40) were then compared with the mean expression levels of the housekeeping genes (*β*-actin). Results that are significantly different (*P* < 0.01) from heart are indicated with ∗∗ and those significantly different (*P* < 0.05) from heart are indicated with ∗. Testis (TE), epididymis (EP), heart (HE), liver (LI), spleen (SP), lung (LU), kidney (KI), muscle (MU), cerebrum (CE), stomach (ST), and small intestine (SM).

The mRNA expression levels of HSPB9 and HSPB10 in adult goat tissues were shown as in Figure 1. The two sHsps both exhibited the highest expression levels in testis. The HSPB9 levels were approximately 20-fold higher than those in other tissues, and HSPB10 levels were 75-fold higher than those in other tissues (*P* < 0.01), respectively. Interestingly, the expression level of HSPB10 in epididymis was also significantly higher than that of other tissues (Figure 1(b)).

### 3.2. Developmental Expression of HSPB9 and HSPB10 mRNA in Testes

**Figure 2:**
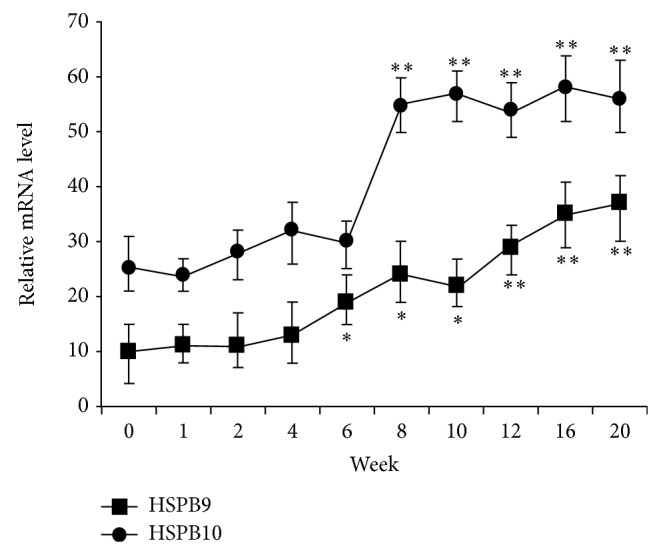
mRNA expression profile of HSPB9 and HSPB10 in different developmental stages of the goat testis. Five independent reactions were performed per sample, and the mean values of 4 samples (mean ± SD, *n* = 20) were then compared with the mean expression levels of the housekeeping genes (*β*-actin). Results that are significantly different (*P* < 0.01) from 0 weeks old are indicated with ∗∗ and those significantly different (*P* < 0.05) from 0 weeks old are indicated with ∗.

mRNA expression profile of HSPB9 and HSPB10 was quantified by qRT-PCR analysis. With the development of testis, the expression level of HSPB9 and HSPB10 showed an increasing trend (Figure 2). Transcript level of HSPB10 was increased suddenly during the later stages (8-wk-old) of testis development and then was maintained at a constant level. The expression level of HSPB9 was increased earlier and slower than that of HSPB10.

### 3.3. Seasonal Changes of HSPB9 and HSPB10 mRNA in Testis

**Figure 3:**
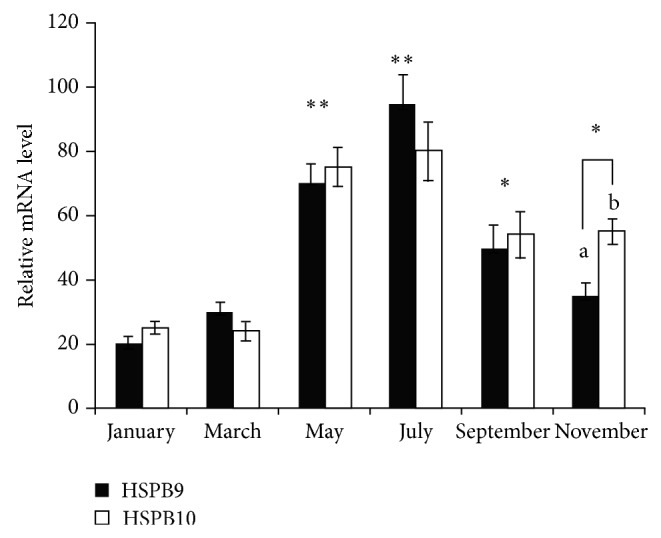
mRNA expression profile of HSPB9 and HSPB10 in different seasons of the goat testis. Five independent reactions were performed per sample, and the mean values of 5 samples (mean ± SD, *n* = 25) were then compared with the mean expression levels of the housekeeping genes (*β*-actin). Results that are significantly different (*P* < 0.01) from January are indicated with ∗∗ and those significantly different (*P* < 0.05) from January are indicated with ∗. Column with the different lowercase letter means significant difference at *P* < 0.05.

qRT-PCR analysis was used to detect the relative transcript levels of HSPB9 and HSPB10 over a period of 12 months in goat testis (Figure 3). The most significant differences were found between spring-summer (March–July) and summer-autumn-winter (July–September–November). The maximal level of HSPB9 and HSPB10 expression was measured in May and July, which met with the hottest season in Hainan Province (*P* < 0.01). In the breeding season (September–November), the mRNA transcript levels of HSPB10 and HSPB9 were higher (*P* < 0.05) than those in sexually inactive season. In addition, discrepancy between mRNA expression of HSPB10 and HSPB9 was also detected in November.

### 3.4. Expression of Hsps in Response to Different Circumstance Temperature

**Figure 4:**
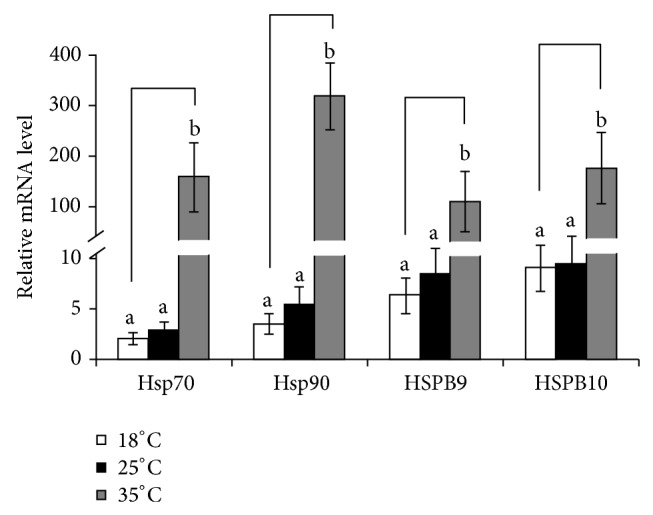
mRNA expression profile of Hsp70, Hsp90, HSPB9, and HSPB10 in response to different environment temperature. Five independent reactions were performed per sample, and the mean values of 5 samples (mean ± SD, *n* = 25) were then compared with the mean expression levels of the housekeeping genes (*β*-actin). Column with the different capital letter means significant difference at *P* < 0.01, and the lowercase letter means significant difference at *P* < 0.05.

As shown in Figure 4, four Hsps had similar expression patterns in responding to the different circumstance temperatures. All four Hsps exhibited the highest expression levels when the goats were exposed at 35°C for 96 h (*P* < 0.01), and the mRNA expression levels of Hsp70, Hsp90, HSPB9, and HSPB10 have dramatically increased about 81-fold, 85-fold, 19-fold and 27-fold, respectively. Hsp70 and Hsp90 were intensively upregulated by heat (*P* < 0.01). There was no remarkable difference among the four Hsps expression at the normal temperature.

### 3.5. Immunohistochemistry Staining of HSPB9 and HSPB10 in Gonad

**Figure 5:**
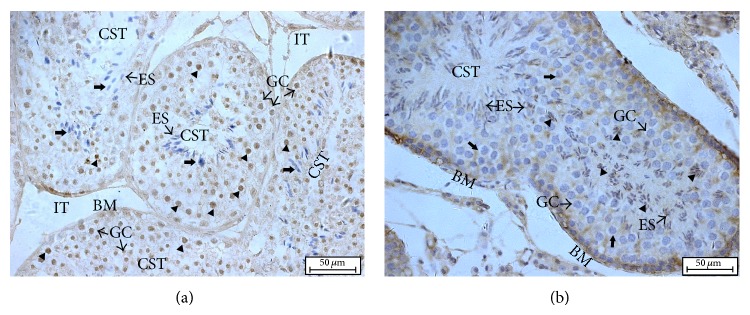
Immunohistochemistry analysis of HSPB9 and HSPB10 in goat testis. The staining was visualized using DAB (brown), and slides were counterstained with hematoxylin. (a) Immunohistochemistry staining result of HSPB9 in goat testis. (b) Immunohistochemistry staining result of HSPB10 in goat testis. HSPB9 is expressed in spermatogonia, spermatocytes, and round spermatids and HSPB10 is expressed in the elongate spermatids. Triangle means the positive staining signal, and arrow shows the negative result. Convoluted seminiferous tubules (CST), interstitial tissue (IT), basement membranes (BM), germ cells (GC), and elongated spermatids (ES).

To determine the allocation of HSPB9 and HSPB10 in gonad, immunohistochemistry staining was performed by using anti-HSPB9 and anti-HSPB10 antibodies. In the normal adult goat testis, spermatogonium, spermatocyte, and spermatid in convoluted seminiferous tubule detected the expression signals of HSPB9. There was no expression signal of HSPB9 in the elongated spermatids (Figure 5(a)). Comparatively, the staining of HSPB10 was mostly restricted to the elongated spermatids. No positive signal of HSPB10 was observed in spermatogenic cells (Figure 5(b)).

**Figure 6:**
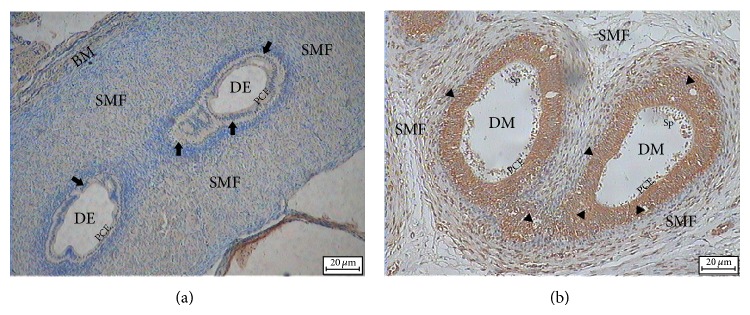
Immunohistochemistry analysis of HSPB9 and HSPB10 in goat epididymis. The staining was visualized using DAB (brown), and slides were counterstained with hematoxylin. (a) Immunohistochemistry staining result of HSPB9 in goat epididymis. (b) Immunohistochemistry staining result of HSPB10 in epididymis. HSPB10 located in the pseudostratified columnar epithelium. Triangle means the positive staining signal, and arrow shows the negative result. Basement membranes (BM), ductus epididymis (DE), pseudostratified columnar epithelium (PCE), smooth muscle fibers (SMF), and spermatozoa (Sp).

In epididymis, there was an obvious difference between the expression of HSPB9 and HSPB10 (Figure 6). HSPB10 was located in pseudostratified columnar epithelium (PCE) of ductus epididymis, and weak immunohistochemistry staining was also observed in smooth muscle fibers (Figure 6(b)). The expression of HSPB9 could not be detected in epididymis samples. The results of immunohistochemistry agreed well with the qRT-PCR data.

## 4. Discussion

Until now, at least 20 heat shock proteins or heat shock factors have been identified as being related to male spermatogenesis [22, 23]. The Hsp70 and Hsp90 are the most widely studied families in these Hsps. Both perform the typical heat inducible chaperone functions, but most of which are also found with impacts on male reproduction. Dix reported that Hsp70 knock-out (KO) resulted in failed meiosis in mice for the first time [24]. Accumulating evidence suggests that Hsp70 may affect male fertility [25, 26]. The association between male fertility and Hsp90 has demonstrated that Hsp90 played important roles in formation of functional forms for steroid hormone receptor [27] and regulating spermatogonia apoptosis [28]. For the typical member of HSPB family, well-studied HSPB1 has been shown to be involved in spermatogenesis [29, 30]. There is little literature focused on gene expression of HSPB9 and HSPB10 in male gonad. So, the main purpose of the present study is to characterize the expression pattern of the two testis-specific HSPB in goat.

As previously reported for other species, HSPB9 and HSPB10 were closely restricted to testis [12, 13, 16, 31]. In the present study, we detected mRNA expression abundance among different tissues of adult rams. In agreement with those previous studies, qRT-PCR results showed that goat HSP9 and HSPB10 were highly expressed in testis but were low or almost absent in spleen, kidney, small intestine, lung, heart, liver, or muscle. Interestingly, expressing HSPB10 was observed to have a high level in epididymis compared with HSPB9. It is possible that this difference may be related to the rigid junction of sperm head and tail for HSPB10.

The presence of different sHsps in a single tissue probably reflects the specific need of that tissue at a certain moment. Well-studied HSPB1 is known to be related to the morphogenesis and cytodifferentiation processes of teeth [32], epidermis [33], skeletal muscles [34], bones, and other tissues [35]. During development of the pig, HSPB family (B-crystallin, Hsp27, cvHsp, and HspB8) displays diverse expression patterns with the age [36]. Based on semiquantitative analysis of other sHsp mRNA in porcine, it is also very likely that the expression levels of HSBP9 and HSPB10 are not constant. In our study, the relative mRNA expression levels of HSBP9 and HSPB10 in testis showed a tendency to increase with age. In this respect, it would be interesting to find out if the sharp increase in expression of HSPB9 and HSPB10 in the testis at 2 months of age is due to spermatogenesis induced by increasing germ cell. However, as complicated regulation of hormone or gene in spermatogenesis, further investigations are needed to determine the mechanism of age-dependent expression for HSPB9 and HSPB10.

Goats have a breeding/reproductive activity that can be considered strongly seasonal. The seasonal characteristics of reproduction tend to become more and more expressed when moving in northerly direction from 35th degree of latitude. In this study, experimental goats reared close to the 20th parallel of north latitude. It was supposed to be that the sexual performance of rams had limited effect on the seasonal changes according to the early studies [37]. Our data clearly showed that the transcript of HSPB9 and HSPB10 was significantly (*P* < 0.05) higher during September to November, while the lower expression levels were measured in the sexually inactive period (during January to March). The tendency is consistent with the seasonal cycle of testicular activity, which was monitored and defined by testicular circumference and testosterone concentration [38]. It seems that such increasing expression of HSPB9 and HSPB10 in breeding season may be associated with male reproductive performance but independent of heat stress. Interestingly, the highest relative expression levels of HSPB9 and HSPB10 were detected around May to July (*P* < 0.01), which overlap with the hottest season of local climate. Therefore, it is supposed that HSPB9 and HSPB10 may be involved in response to circumstance heat stress. In order to determine whether the two testis-specific sHsps responded to the high environment temperature, rams were exposed to different temperature. As expected, two classical Hsps (Hsp70 and Hsp90) and two sHsps (HSPB9 and HSPB10) were upregulated with the increased temperature. Although there has only been one report on the upregulation of HSPB9 by heat shock [39], it was expected that there would be a positive relation between the expression of sHsps and heat tolerance in testis. Unfortunately, one-to-one yeast two-hybridization study was not carried out to further determine whether the sHsps interacted with each other directly.

Spermatogenesis in the convoluted seminiferous tubules can be divided into three different phases: the proliferative phase in which spermatogonia divide, the meiotic phase in which genetic material in spermatocytes is recombined and segregated, and the spermiogenic phase in which round spermatids transform through elongate spermatids into spermatozoa. In situ hybridization studies have demonstrated that HSPB9 was localized in diplotene and stage XII (meiosis I and meiosis II) spermatocytes, round spermatids, and steps 9~12 elongate spermatids. No signal was found in other types of germ cells [13]. However, de Wit et al. reported that HSPB9 staining was detected in the nuclei of spermatogonia by immunohistochemistry studies [12]. In the present study, immunohistochemistry staining results clearly showed that HSPB9 was expressed in spermatogonia, spermatocytes, and round spermatids. Elongate spermatids into spermatozoa, Sertoli cells, and pseudostratified columnar epithelium in epididymis were all negative. Hsp27 was allocated in spermatocytes, spermatogonia, spermatids, and Sertoli cells. This discrepancy can be used to distinguish HSPB9 and Hsp27. It has been reported that loss of HSPB10 did not affect female mice, and impairment of HSPB10 even caused male infertility [16]. Unsurprisingly, HSPB10/ODF1 is exclusively expressed in spermatozoa by immunohistochemistry analysis. Highly expressed HSPB10 in pseudostratified columnar epithelium was also verified by the qRT-PCR result. It is worth mentioning that strong staining of HSPB10 was observed in epididymis. Storage and maturation of sperm take place in epididymis. Different kinds of secretory proteins are believed to be involved in this process. Eickhoff reported that some of the secretory proteins (cytokine macrophage migration inhibitory factor/MIF) were identified as a new component of the outer dense fibers [40]. It seems to explain the reason why the high expression level of HSPB10 was detected in epididymis. However, there is no further study to support this deduction.

In summary, the present study demonstrated that the transcript levels of HSPB9 and HSPB10 were extremely high in the testis. The relative mRNA expression of two sHsps showed a tendency to increase with age. An increasing expression level of HSPB9 and HSPB10 was also found in breeding season. Both HSPB9 and HSPB10 were found to also be upregulated by high temperature. High HSPB9 expression was found in spermatogenic cells, including spermatogonium, spermatocyte and spermatid in convoluted seminiferous tubule, and high HSPB10 expression in elongated spermatids, respectively. The temporal-specific and tissue-specific expressed HSPBB9 and HSPB10 may be closely related to male reproduction and heat tolerance. These details are suggested to be providing valuable data for further studies of the mechanism of HSPB9 and HSPB10 in regulating spermatogenesis and heat stress.
